# Exosome-mediated targeted delivery of miR-210 for angiogenic therapy after cerebral ischemia in mice

**DOI:** 10.1186/s12951-019-0461-7

**Published:** 2019-02-19

**Authors:** Huixin Zhang, Jin Wu, Jiahuan Wu, Qi Fan, Jingchao Zhou, Junwen Wu, Sichen Liu, Jie Zang, Jinhai Ye, Ming Xiao, Tian Tian, Jun Gao

**Affiliations:** 1 The Department of Neurobiology, Key Laboratory of Human Functional Genomics of Jiangsu, Nanjing Medical University, Nanjing, 211166 Jiangsu China; 2 The Department of Neurology, The Second Affiliated Hospital of Nanjing Medical University, Nanjing, 210011 Jiangsu China; 3 The School of Basic Medical Science, Nanjing Medical University, Nanjing, 211166 Jiangsu China; 4 The Department of Oral and Maxillofacial Surgery, The Affiliated Stomatology Hospital of Nanjing Medical University, Nanjing Medical University, Nanjing, 210029 China; 5 Jiangsu Key Laboratory of Neurodegeneration, Nanjing Medical University, Nanjing, 211166 Jiangsu China

**Keywords:** Ischemia, miR-210, Exosomes, Angiogenesis

## Abstract

**Background:**

Accumulating evidence shows that microRNA-210 (miR-210) holds great promise to improve angiogenesis for brain tissue repair after cerebral ischemia. However, safe and efficient delivery of miR-210 via intravenous administration is still a challenge. In the past decade, exosomes have emerged as a novel endogenous delivery system. Here, c(RGDyK) peptide is conjugated to exosomes, and they are loaded with cholesterol-modified miR-210 (RGD-exo:miR-210).

**Results:**

In a transient middle cerebral artery occlusion (MCAO) mouse model, the RGD-exo:miR-210 targets the lesion region of the ischemic brain after intravenous administration, resulting in an increase in miR-210 at the site. Furthermore, RGD-exo:miR-210 are administered once every other day for 14 days, and the expressions of integrin β_3_, vascular endothelial growth factor (VEGF) and CD34 are significantly upregulated. The animal survival rate is also enhanced.

**Conclusions:**

These results suggest a strategy for the targeted delivery of miR-210 to ischemic brain and provide an angiogenic agent for the treatment of ischemic stroke.

**Electronic supplementary material:**

The online version of this article (10.1186/s12951-019-0461-7) contains supplementary material, which is available to authorized users.

## Background

Ischemic stroke is a major global disease associated with high morbidity, mortality, and healthcare expenditure. However, the effective clinical therapy for stroke is still lacking [1]. Recently, an extensive number of investigations have concentrated on poststroke vascularization because functional recovery is highly dependent on the effective restoration of blood supply [2–4]. MicroRNAs (miRNAs), small noncoding RNAs, have critical roles in regulating gene expression and hold great promise for enhancing the efficiency of angiogenesis for stroke treatment given their ability to regulate multiple targeted genes and their small size [5–7].

MiR-210 is the master hypoxia-induced miRNA (hypoxia-miR) and promotes angiogenesis mediated by the vascular endothelial growth factor (VEGF) signaling pathway [8–10]. Accumulating evidence shows that overexpression of miR-210 upregulates focal angiogenesis and improves functional recovery in ischemia/reperfusion models, including middle cerebral artery occlusion (MCAO) [11], myocardial infarction [12], renal ischemia [13] and hindlimb ischemia models. Specifically, it has been reported that injected lentiviral vector can carry miR-210 transcranially into ischemic mouse brain, resulting in focal angiogenesis and improvement of neurobehavioral outcomes [14, 15]. However, intravenous administration is not an acceptable route for clinical application. Hence, a safe and efficient delivery system that can cross the blood-brain barrier (BBB) is critically needed [16, 17].

Exosomes, 40–150 nm extracellular vesicles (EVs) released by cells, have emerged as a novel endogenous delivery system [18–21]. As natural carriers that transfer bioactive molecules between cells, exosomes present several features, including low immunogenicity, biodegradability, ability to encapsulate endogenous bioactive molecules, and the ability to cross the BBB [22–24]. In previous work, to improve the targeting characteristics of exosomes, we developed a rapid and efficient method to conjugate functional ligands onto exosomal surfaces using bio-orthogonal copper-free azide alkyne cyclo-addition (click chemistry) [25]. The cyclo (Arg-Gly-Asp-D-Tyr-Lys) peptide [c(RGDyK)] conjugated exosomes (RGD-exo) have been showed to target the ischemic brain after intravenous administration through binding between c(RGDyK) and integrin α_v_β_3_ in reactive cerebral vascular endothelial cells. Furthermore, to load nucleic acids into exosomes, several strategies have been developed, including electroporation, sonication, incubation with permeabilization agents, and incubation with lipophilically modified RNAs [26, 27]. Moreover, the EVs loaded with cholesterol-conjugated small interfering RNA (siRNA) have been applied for functional silencing of a target gene in cells [28]. Hence, we hypothesized that miR-210-loaded RGD-exo (RGD-exo:miR-210) could deliver miR-210 to the ischemic brain through intravenous injection and induce focal angiogenesis.

Here, we conjugated c(RGDyK) peptide on mesenchymal stromal cell (MSC)-derived exosomes and loaded the exosomes with cholesterol-modified miR-210. Mice were subjected to MCAO and reperfusion (MCAO/R) to produce an ischemic stroke model. Subsequently, RGD-exo:miR-210 were administered intravenously, and near-infrared fluorescence (NIRF) imaging showed their ability to target the ischemic brain. The enhanced miR-210 and VEGF levels in the lesion region indicated that miR-210 was delivered and took effect. Furthermore, RGD-exo:miR-210 were administered once every other day for 14 days, and a significant increase in integrin β_3_, VEGF and CD34 indicated that angiogenesis was improved. Finally, a significant increase in animal survival was evident after treatment with RGD-exo:miR-210.

## Results

### miR-210 expression in the lesion region is influenced by ischemia

**Fig. 1:**
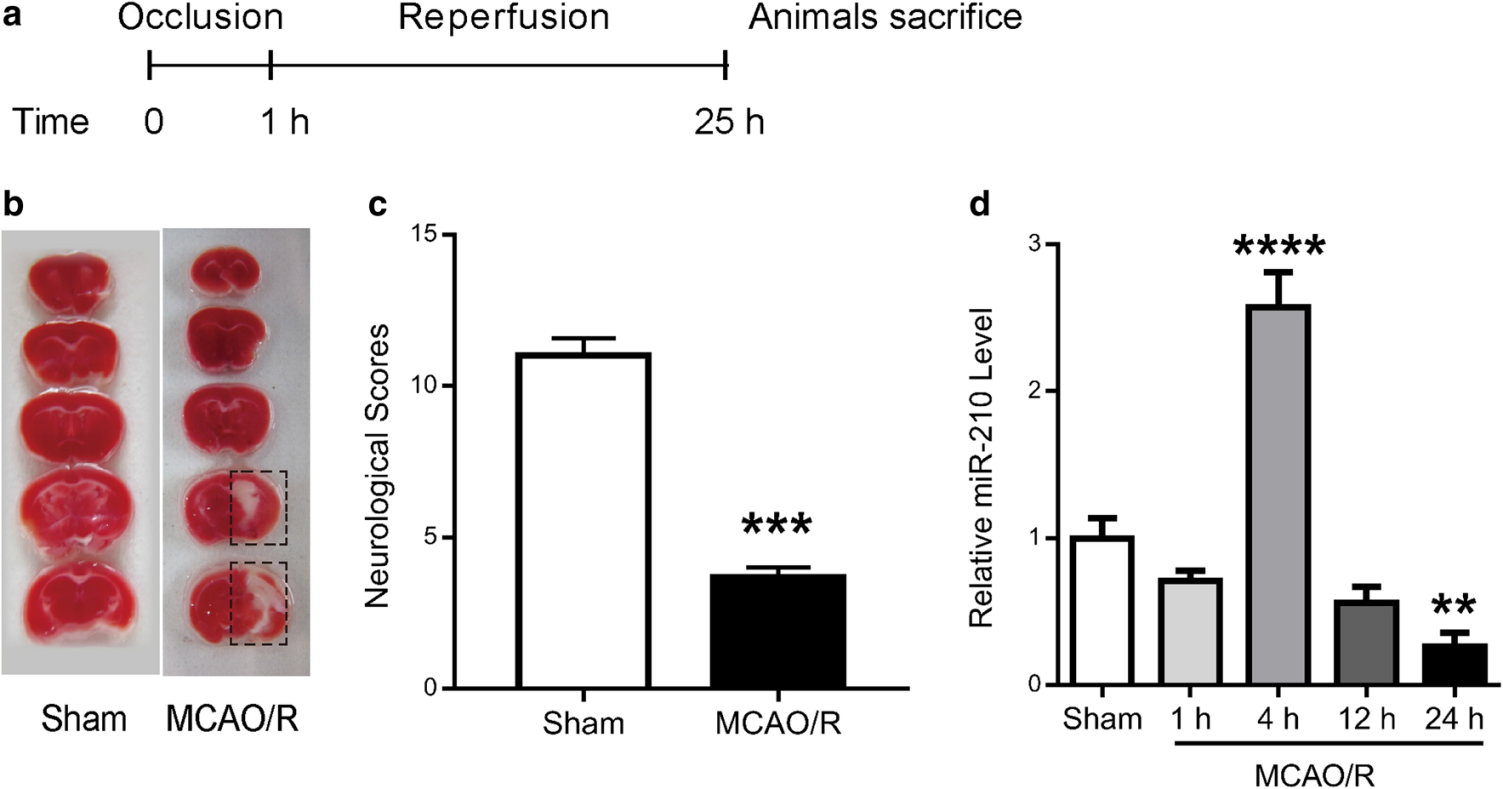
The expression changes in miR-210 after ischemia in vivo. **a** The schematic time course of the animal models developed by inducing ischemia for 1 h and reperfusion for 24 h. **b** TTC staining of 2-mm coronal brain slides; the lesion region is in dashed frames. **c** The neurological scores of mice receiving 1 h of MCAO and 24 h of reperfusion. The sham group was used as the control. N ≥ 3. The data are expressed as the mean ± SEM; ***P < 0.001 by Student’s t-test. **d** Quantitative analysis showing the relative miR-210 levels in the lesion region at different time points after reperfusion or in the sham group. N ≥ 3. The data are expressed as the mean ± SEM; ****P < 0.0001, and **P < 0.01, compared with the sham group by one-way ANOVA

Mice were subjected to MCAO/R in the right hemisphere according to the procedure (Fig. 1a). The representative 2,3,5-triphenyltetrazolium chloride (TTC)-stained brain sections showed infarct areas, and the neurological scores presented the functional deficit after 1 h of MCAO and 24 h of reperfusion (Fig. 1b, c). In this well-established animal model, the lesion region consisted of the lateral striatum, the overlying cortex, and the adjacent ventrolateral neocortex. Following previous reports [25, 29], the typical lesion region is indicated in Fig. 1b (see “Methods” for details).

Previous evidence has confirmed the crucial role of miR-210 in regulation of the cell response to hypoxia or ischemia. To evaluate the miR-210 expression level in our model, the brain tissue corresponding to the lesion region was collected, and quantitative real-time PCR was performed (Fig. 1d). As shown in Fig. 1d, ischemia and reperfusion induced a rapid and significant change in the miR-210 level in the lesion region. Interestingly, this change was time dependent. The miR-210 level remarkably increased 4 h after reperfusion but decreased 24 h after reperfusion. According to the literature, miR-210 inhibition causes severer tissue damage following ischemia [30, 31], whereas miR-210 overexpression shows better outcomes [15]. Thus, we aimed to develop a strategy for promoting recovery through overexpression of miR-210 24 h after reperfusion.

### Conjugation of c(RGDyK) peptide and loading of miR-210 into exosomes

**Fig. 2:**
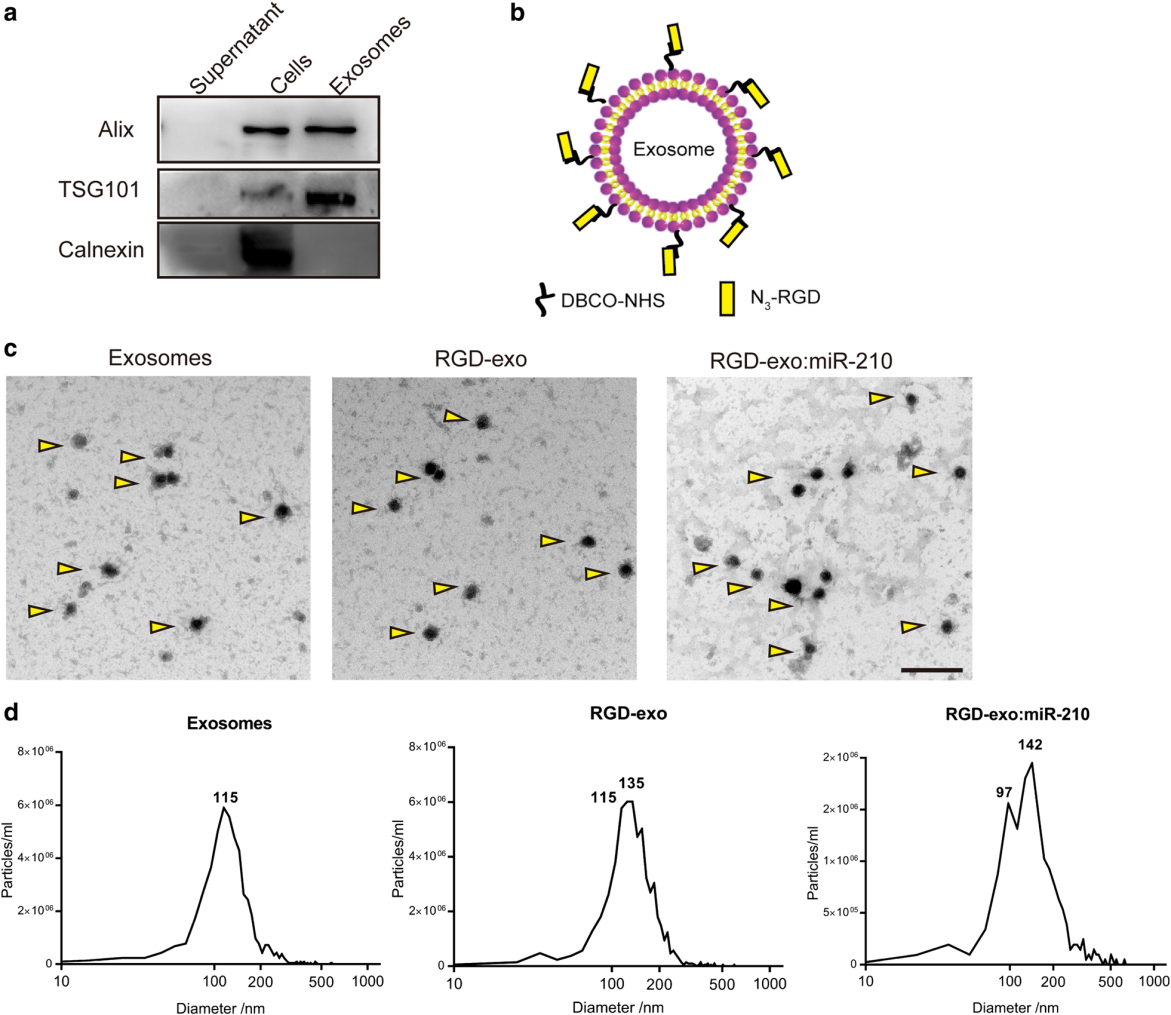
Characterization of unmodified exosomes and the RGD-exo loaded with miR-210. **a** Western blot analysis of Alix, TSG101, and calnexin expression in cells and in exosomes isolated from their conditioned medium. The supernatant obtained from ultracentrifugation during exosome isolation was used as the negative control. **b** Schematic diagram of functionalized exosomes with DBCO groups and c(RGDyK) peptides. **c** Transmission electron micrograph of the exosomes, RGD-exo, and RGD-exo loaded with miR-210 (triangles). Scale bar: 500 nm. **d** Size distribution of unmodified exosomes, the RGD-exo, and the RGD-exo:miR-210 detected by NTA measurements

Intravenous administration of exogenous miR-210 is an acceptable route to induce miR-210 overexpression in ischemic brain. Exosomes show great promise as a safe and efficient vehicle for miR-210 delivery. Bone marrow-derived MSCs were cultured and their conditioned medium was collected for exosome isolation. Western blotting, transmission electron microscopy (TEM), and nanoparticle tracking analysis (NTA) were performed to characterize the exosomes. Western blotting showed that Alix and TSG101, known as representative exosomal markers, were enriched in exosomes, while Calnexin (a negative marker) was not detected (Fig. 2a). To improve the targeting ability toward ischemic brain, c(RGDyK) peptide was conjugated to the exosome surface using bio-orthogonal copper-free click chemistry described in our previous work (Fig. 2b) [25]. To estimate the number of peptides on the exosomes, a fluorescent fluorescein isothiocyanate (FITC)-labeled peptide (c(RK(FITC)DyK)) was used for conjugation. From a fluorescent standard curve of free c(RK(FITC)DyK), we calculated that 500 µg/mL modified exosomes contained 362 nM peptides on average (Additional file 1: Figure S1a, b). Measured by NTA, 500 µg protein of exosomes contained 3.1 × 10^11^ exosome particles approximately. The average peptide density was calculated to be 116 pmol/10^11^ particles. Next, RGD-exo were incubated with cholesterol-modified miR-210, followed by ultracentrifugation. According to previous reports, lipophilic miR-210 can self-associate with exosomes [28, 32]. Using FITC-labeled miR-210 (with cholesterol modification), it was calculated that 500 µg/mL RGD-exo contained 337 nM miR-210 on average. Given that 100 µg/mL exosomes were incubated with 100 nM miR-210 at the beginning, the loading rate is 67.4% approximately. The average density of miR-210 was 108 pmol/10^11^ particles (Additional file 1: Figure S1c, d). As revealed by TEM, both RGD-exo and RGD-exo:miR-210 were round in shape (Fig. 2c). In addition, NTA analysis showed a similar size distribution between unmodified exosomes and RGD-exo and a shift to slightly larger vesicles after miR-210 incorporation, in line with a previous report on vesicle size after loading of cholesterol-conjugated siRNA (Fig. 2d) [28]. These data indicate that conjugation of c(RGDyK) to exosomes and miRNA loading did not alter the basic properties of the exosomes.

### RGD-exo:miR-210 targets ischemic brain after intravenous administration

**Fig. 3:**
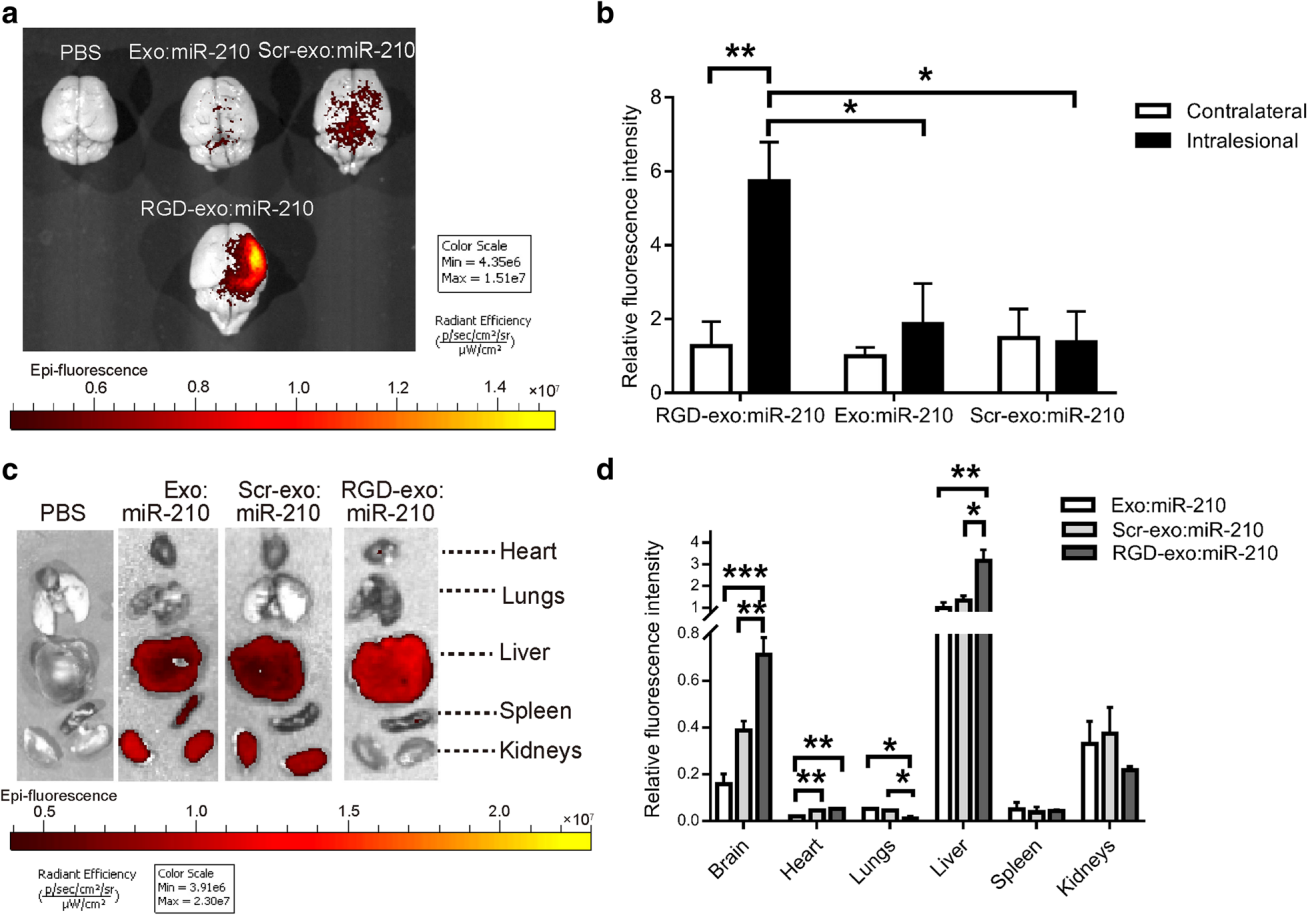
RGD-exo target the lesion region after intravenous administration. **a** Representative NIRF images of mouse brains that received MCAO/R and PBS, exosomes with miR-210 (Exo:miR-210), Scr-exo:miR-210, or RGD-exo:miR-210 administration. Brains were dissected 6 h after administration. **b** Quantitation of fluorescence intensity in the lesion region (intralesional) and matching nonlesion region (contralateral). The data are expressed as the mean ± SEM; *P < 0.05, and **P < 0.01, compared with the contralateral groups by two-way ANOVA. **c** The representative fluorescence distribution in organs (heart, lungs, liver, spleen, kidneys) dissected from mice that received MCAO/R and PBS, Exo:miR-210, Scr-exo:miR-210, or RGD-exo:miR-210 administration. Organs were dissected 6 h after administration. **d** Quantitation of fluorescence intensity in organs. The data are expressed as the mean ± SEM; *P < 0.05, **P < 0.01, and ***P < 0.001 by one-way ANOVA

Our previous work demonstrated the tropism of RGD-exo to the lesion region of the ischemic brain [25]. Here, to evaluate the targeting ability of RGD-exo:miR-210 in vivo, the fluorophore cyanine 5.5 far-red fluorescent dye (Cy5.5) was conjugated to Exo:miR-210, Scr-exo:miR-210, or RGD-exo:miR-210 to visualize their biodistribution using NIRF imaging. By a fluorescent standard curve of free Cy5.5-azide, we estimated 500 µg/mL Exo, Scr-exo, or RGD-exo contained 221, 209, or 202 nM Cy5.5 on average, respectively (Additional file 1: Figure S1e, f). No significant difference is observed among the three groups. Mice subjected to 1 h of MCAO and 24 h of reperfusion were injected with Cy5.5-labeled and miR-210-loaded RGD-exo, Scr-exo, or exosomes via the tail vein. Six hours later, the mice were sacrificed, and their brains were dissected. NIRF imaging showed a significantly higher fluorescence intensity in the lesion region after RGD-exo:miR-210 administration compared with Scr-exo:miR-210 or Exo:miR-210, whereas fewer RGD-exo:miR-210 reached the contralesional region (Fig. 3a, b). Next, other organs were dissected and analyzed via NIRF imaging. As shown in Fig. 3c and d, unmodified exosomes with miR-210 incorporation primarily accumulated in the liver, followed by the kidneys. RGD-exo:miR-210 administration increased the signal in the brain and liver. These data were in line with a previous study on RGD-exo without RNA loading [25], indicating that miR-210 loading did not affect the targeting ability of RGD-exo in the MCAO/R model.

**Fig. 4:**
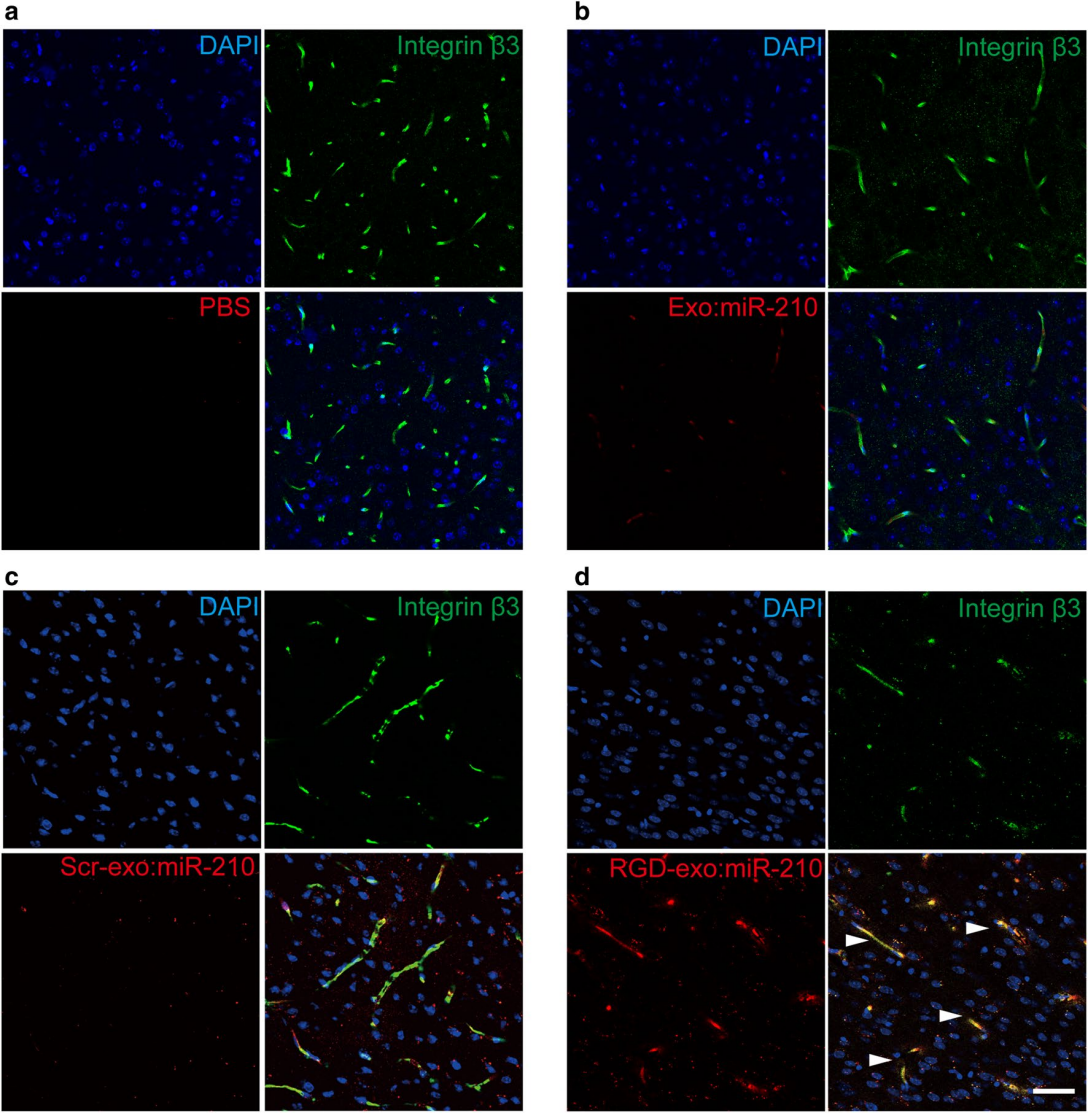
RGD-exo:miR-210 targets integrin β_3_ in the lesion region after intravenous administration. **a**–**d** Co-labeled fluorescence images of PBS, Exo:miR-210, Scr-exo:miR-210 or RGD-exo:miR-210 (red) with integrin β_3_ (green) in the ischemic cortex 6 h after intravenous administration of tdTomato-labeled exosomes. Blue indicates nuclei. The triangles indicate the co-localization of RGD-exo and integrin β_3_. Scale bar: 50 µm

To further examine the targeting mechanism, brains were sectioned and immuno-stained for integrin β_3_ and CD34 (a marker of endothelial cells). Following previous work, the exosome membrane was labeled by fusing tdTomato with a palmitoylation signal [33]. As revealed by confocal imaging, after MCAO/R, RGD-exo:miR-210 strongly co-localized with integrin β_3_ in the lesion region 6 h after intravenous administration (Fig. 4d). In contrast, the signals after the injection of phosphate-buffered saline (PBS), Exo:miR-210 or Scr-exo:miR-210 was minimal (Fig. 4a–c). In addition, obvious co-localization was detected between RGD-exo and CD34 in the ischemic brain (Additional file 2: Figure S2). These data were consistent with previous studies [25, 34]. There is only one integrin β_3_, integrin α_v_β_3_ expresses in brain endothelial cells [35, 36]. The targeting ability resulted from the affinity between c(RGDyK) and integrin α_v_β_3_ which is related to the angiogenic response to brain ischemia.

### miR-210 takes effects in ischemic brain when delivered by RGD-exo

**Fig. 5:**
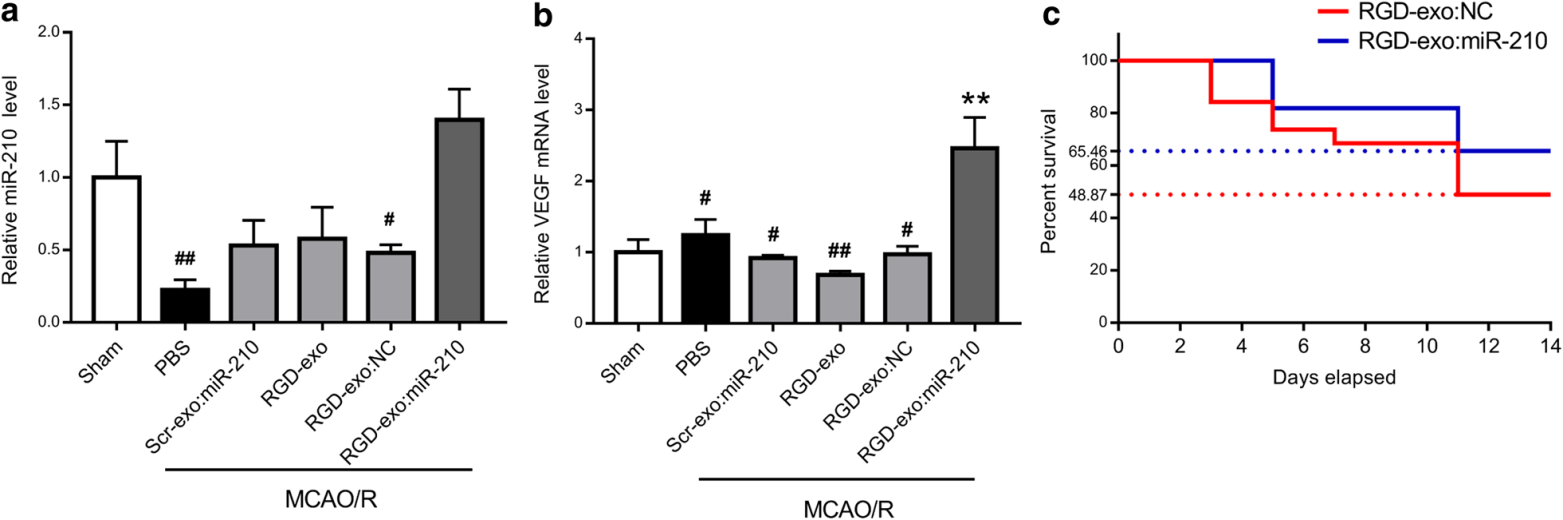
miR-210 and VEGF levels in the lesion region were upregulated by administration of RGD-exo loaded with miR-210 and alleviated the mortality after MCAO/R. **a** Intravenous administration of PBS, Scr-exo:miR-210, RGD-exo, RGD-exo loaded with negative control (RGD-exo:NC), or RGD-exo loaded with miR-210 (RGD-exo:miR-210) was performed on mice subjected to 1 h of MCAO and 24 h of reperfusion. miR-210 in the lesion region was analyzed 12 h after administration (36 h after reperfusion). N ≥ 3. The data are expressed as the mean ± SEM. ^#^P < 0.05, and ^##^P < 0.01 by one-way ANOVA. **b** The mRNA level of VEGF was examined 24 h after administration (48 h after reperfusion). The sham group was used as the control. N ≥ 3. The data are expressed as the mean ± SEM. **P < 0.01 compared with sham, ^#^P < 0.05 and ^##^P < 0.01 compared with RGD-exo:miR-210 by one-way ANOVA. **c** The survival percentages of the mice that received MCAO/R followed by RGD-exo:NC or RGD-exo:miR-210 administration

To confirm the delivery of miR-210 to ischemic brain after intravenous injection of RGD-exo:miR-210, the brain tissue corresponding to the lesion region was dissected for quantitative PCR analysis. An enhanced level of miR-210 was found 12 h after RGD-exo:miR-210 administration compared with the level following PBS, Scr-exo:miR-210, RGD-exo, or negative control (RGD-exo:NC) treatments (Fig. 5a). According to the literature, the therapeutic potential of miR-210 is mediated by modulation of multiple downstream factors, including the VEGF signaling pathway [9, 15]. Thus, we evaluated the mRNA level of VEGF in the lesion region 24 h post-injection (Fig. 5b). As a result, RGD-exo:miR-210 upregulated VEGF more effectively than Scr-exo:miR-210, RGD-exo:NC, RGD-exo or PBS. These results demonstrated that exogenous miR-210 was delivered to the lesion region and exerted its effects.

Next, to examine the therapeutic potential of RGD-exo:miR-210 for ischemic brain, a 30 min MCAO was performed to produce a moderate ischemia, allowing study of the long-term outcome. After 24 h of reperfusion, RGD-exo:miR-210 or RGD-exo:NC were administered once every other day. Fourteen days later, a significant increase in animal survival was evident with RGD-exo:miR-210 treatment compared with animals treated with RGD-exo:NC (Fig. 5c, 65.46% for RGD-exo:miR-210, 48.87% for RGD-exo:NC). Thus, systemic administration of RGD-exo:miR-210 was effective against cerebral ischemia.

### RGD-exo:miR-210 promotes VEGF expression and angiogenesis

**Fig. 6:**
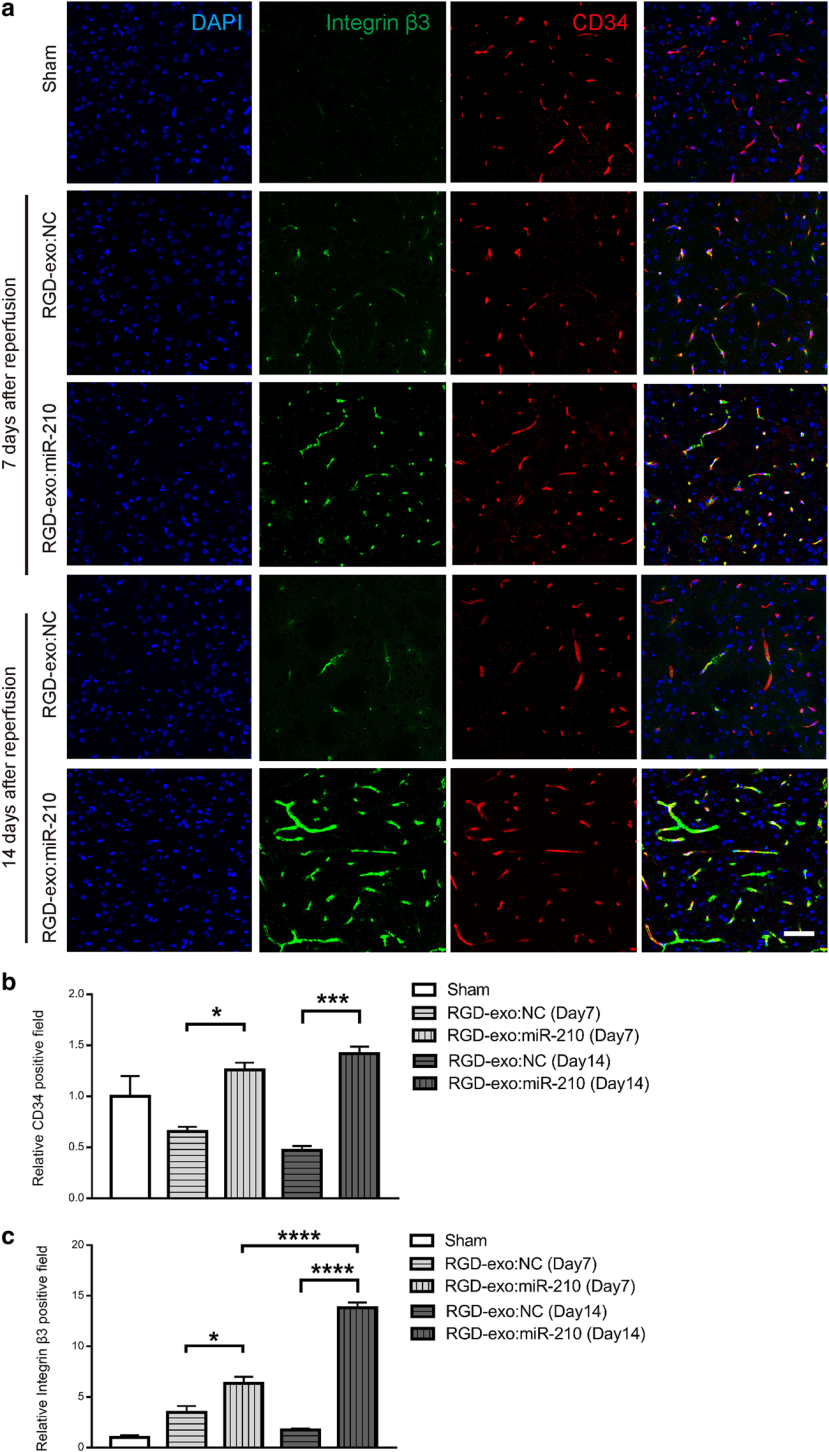
Up-regulation of integrin β_3_ and CD34 expression in the lesion region after administration of RGD-exo:miR-210. **a** Immunofluorescence images of integrin β_3_ and CD34 in the lesion region 7 or 14 days after reperfusion. The sham group was used as the control. Scale bar: 50 µm. **b**, **c** Quantitation of CD34 and integrin β_3_ densities in the lesion region. The data are expressed as the mean ± SEM. *P < 0.05, ***P < 0.001, and ****P < 0.0001 by one-way ANOVA

**Fig. 7:**
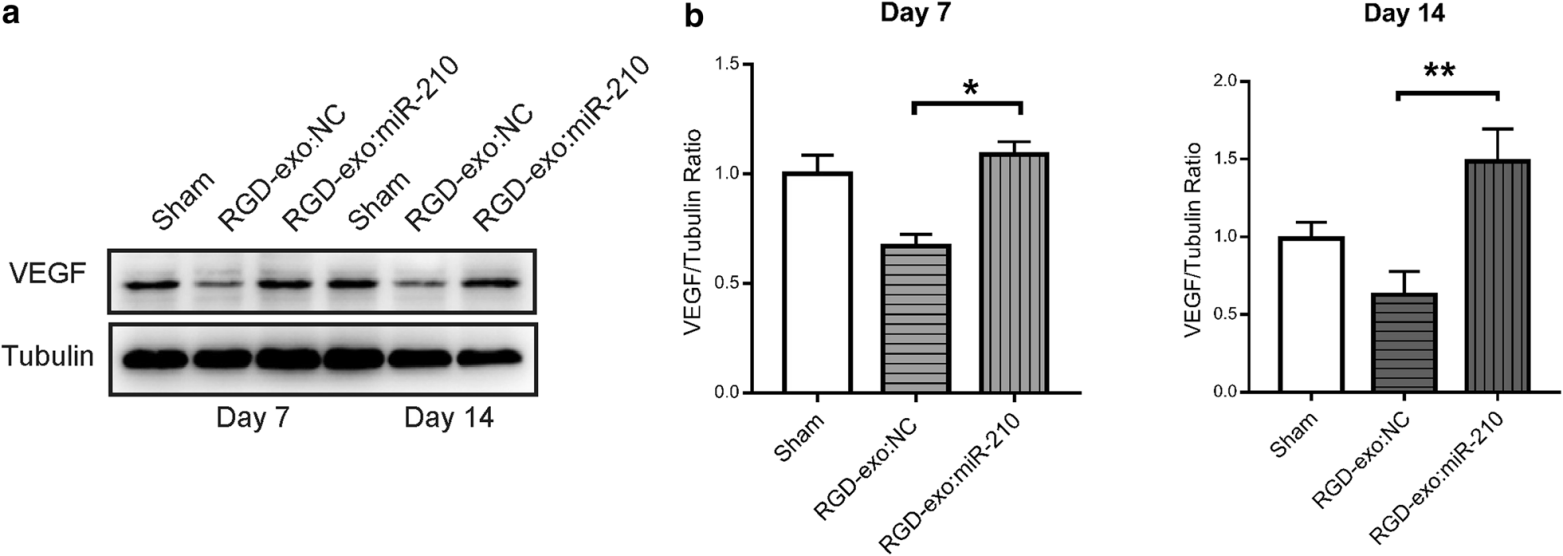
RGD-exo:miR-210 administration promoted angiogenesis in the ischemic brain. **a** Analysis of VEGF expression in the lesion region 7 or 14 days after reperfusion and administration of RGD-exo:NC or RGD-exo:miR-210. **b** Quantitation of VEGF expression 7 or 14 days after reperfusion and administration. N ≥ 3. The data are presented as the mean ± SEM. *P < 0.05 and **P < 0.01 by one-way ANOVA

To determine whether RGD-exo:miR-210 induced angiogenesis, CD34 was imaged and quantitatively analyzed. The microvessel density (CD34^+^/field) increased in the lesion region in the mice receiving RGD-exo:miR-210 compared with those receiving RGD-exo:NC after 7 and 14 days of treatment (Fig. 6a, b). Interestingly, integrin β_3_ was also upregulated by RGD-exo:miR-210 administration (Fig. 6a, c). It is known that integrin α_v_β_3_ is involved in the angiogenesis pathway initiated by VEGF. Thus, the above data indicate that miR-210 induced angiogenesis in the lesion region. Meanwhile, a strong co-localization between integrin β_3_ and CD34 was observed over time in the mice treated with RGD-exo:miR-210, resulting in the binding site on the endothelial cells for RGD-exo being maintained for at least 14 days in the lesion region. Furthermore, VEGF, the downstream factor of miR-210, was detected by Western blotting (Fig. 7a, b). In RGD-exo:miR-210 treated mice, the VEGF level was significantly enhanced compared with that in mice receiving RGD-exo:NC. VEGF is a well-known specific mitogen of endothelial cells which induces their proliferation [37]. To provide experimental evidence, co-labeled immunostaining of 5-bromo-2-deoxyuridine (BrdU, an indicator of proliferation) and CD34 was performed on MCAO/R mice at 7 days after reperfusion. As shown in Additional file 3: Figure S3, significantly more BrdU^+^ endothelial cells are observed after RGD-exo:miR-210 injection compared with that of RGD-exo:NC treatment. This result indicated endothelial cell proliferation is involved in miR-210-promoted angiogenesis.

## Discussion

In this study, we proposed a strategy to deliver therapeutic nucleic acids to ischemic brain. Specifically, VEGF expression and angiogenesis in the lesion region was enhanced by intravenous administration of RGD-exo:miR-210. miR-210 plays multiple crucial roles in the cellular regulation response to low oxygen, including ischemic brain injury. In addition to improving angiogenesis, miR-210 can also inhibit apoptosis, support stem cell survival, and repress mitochondrial metabolism [38, 39]. Previous studies have shown that miR-210 protects against hypoxia-induced apoptosis by targeting the HIF-1α pathway and increases adipose-derived stem cell (ASC) proliferation and migration via iron–sulfur cluster scaffold homolog 2 (ISCU2) and protein tyrosine phosphatase, nonreceptor type 2 (PTPN2), leading to different therapeutic effects according to the hypoxia level [40, 41]. Hence, miR-210 has been proposed as a potential therapeutic target. Brain delivery of lentiviral miR-210 enhanced microvessel density and improved neurobehavioral outcomes in ischemic mouse. Clinical studies have demonstrated that miR-210 levels are significantly higher in stroke patients with good outcomes than in those with poor outcomes. Indeed, we found that the vascular density increases and remains high for 2 weeks after RGD-exo:miR-210 administration. In addition, the relationship between angiogenesis and neurogenesis after cerebral ischemia has been widely studied [42]. Consistent with previous reports, a significant increase in animal survival was found with RGD-exo:miR-210 treatment.

Angiogenic therapy with miR-210 is impeded by challenges in delivery across the BBB to the ischemic brain. Exosomes are believed to be a potential delivery vehicle, given their unique properties, including low immunogenicity, biodegradability, low toxicity, strong protection for cargo and the ability to cross the BBB [43]. Our previous study showed that RGD-exo can act as a drug carrier that targets the ischemic brain. In previous reports, intravenous infusion of MSC-derived exosomes has been confirmed to be well tolerated and beneficial for stroke recovery [44, 45], and thus, here, exosomes were isolated from the conditioned medium of MSCs and conjugated with c(RGDyK). For loading of miR-210 into exosomes, several strategies have been developed, including electroporation, sonication, incubation with permeabilization agents, and incubation with lipophilically modified RNAs. It has been reported that electroporation can induce precipitation and aggregation of the siRNA, leading to overestimation of vesicle loading [46]. In addition, sonication and incubation with permeabilization agents can cause a reformation/deformation of exosomes to disrupt exosome integrity [27]. Due to the difficulties in loading, attempts have been made recently to load EVs with hydrophobically modified RNAs. Recent study has described the method to load EVs with cholesterol-conjugated siRNA for functional dose-dependent silencing of the target gene human antigen R, as a potential drug target to reduce tumor growth [28]. Besides, the cholesterol enables quick membrane association, for the single-stranded phosphorothioated tail is essential for cellular internalization by a mechanism similar to that used by conventional antisense oligonucleotides, which can be easily controlled or scaled for production [32]. In this work, although a shift in size distribution to slightly larger vesicles was observed following incubation with cholesterol-modified miR-210, the shape of the size distribution curve of RGD-exo remained constant. The results are consistent with a previous report on loading EVs with siRNA, suggesting that the integrity of exosomes was not affected. Moreover, the targeting ability of RGD-exo:miR-210 was confirmed by NIRF imaging and immunofluorescence, indicating that the incorporation of cholesterol-modified miR-210 did not affect the tropism of RGD-exo to ischemic brain.

After intravenous administration, RGD-exo:miR-210 bind integrin α_v_β_3_ on reactive cerebral vascular endothelial cells in the ischemic area. Then exosomes can enter recipient cells through different types of endocytosis [47]. Once involved into endosomal system, exosomes can fuse with the organelle membrane and release their contents into cytoplasma [48]. Thus, RGD-exo:miR-210 hold the potential to deliver exogenous miR-210 to endothelial cells in the lesion region. To observe long-term outcomes, a milder ischemia model (0.5 h MCAO and 24 h reperfusion) was used. RGD-exo:miR-210 were injected once every other day via the tail vein. Generally, integrin β_3_, the binding site on endothelial cells for RGD-exo, reaches its peak on day 10 after cerebral ischemia and then significantly decreases [2, 3]. Interestingly, the treatment with miR-210 strongly upregulated integrin β_3_ expression on day 7 compared with NC treatment, followed by higher expression of integrin β_3_ on day 14 induced by miR-210. Thus, the improved angiogenesis provided more affinity sites for c(RGDyK) to bind, resulting in maintenance of the targeting ability of RGD-exo for at least 14 days. Thus, in the future, miR-210 co-incorporation could be a strategy to extend the time window for delivering other therapeutic agents to ischemic brain through RGD-exo.

Together, RGD-exo were shown to be a robust vehicle for targeted delivery of miR-210 to ischemic brain via intravenous administration. Other functional miRNAs or several miRNAs together could be loaded into RGD-exo in the future. RGD-exo:miR-210 were shown to be a promising therapeutic agent for neural protection by promoting angiogenesis and prolonged the time window for RGD-exo targeted delivery. We believe that other therapeutic agents could be co-incorporated into RGD-exo with miR-210 for clinical application in ischemic disease.

## Conclusions

In conclusion, we have developed a potent, efficient and effective strategy based on RGD-exosome loaded with miR-210 inducing an accumulation of miR-210 in lesion region which is downregulated in ischemia, to promote microvascular angiogenesis. The modified exosome vehicle can help to overcome a target transport through BBB, and attenuate stroke symptoms by preventing miRNA degradation, consistent with the protein VEGF up-regulating. Our findings could be a promising therapeutic strategy in biological delivery for neural system protection.

## Methods

### Animals

The mice were purchased from The Animal Core Facility of Nanjing Medical University (Nanjing, China). All the animal experiments were carried out in accordance with the Animal Care and Use Committee of Nanjing Medical University (no. IACUC-1807005). Eight- to 10-week-old male C57BL/6 mice were used. Animals were group housed at a controlled temperature (20 ± 2 °C) with a 12 h light–dark cycle and free access to food and water.

### The ischemic stroke model and lesion region demarcation

For the MCAO/R model in mice, following a previous study [49], after animal anesthesia a midline neck incision was made to expose both common carotid arteries (CCAs). After anterior and downward retraction of the musculature, the right CCA was temporarily occluded with a microaneurysm clip, and a cut was made along the external carotid arteries. Then, middle carotid artery (MCA) occlusion was induced by inserting a silicon-coated 6-0 nylon filament along the internal carotid artery with the aid of an operating microscope and advancing the filament 8–10 mm distally. Reperfusion was induced at a certain timepoint by monofilament removal according to the experiment. In addition, a 75–90% blood flow decrease in the MCA territory was recorded by laser Doppler flow during each experiment after reperfusion using a flexible probe attached to the animals’ skull. The mice were kept warm at 37 °C with a heated blanket throughout the surgery and during recovery from anesthesia and then returned to their home cages. Sham-operated mice were subjected only to exposure of the MCA without ischemia induction. The lesion region is described and shown in Fig. 1b. Briefly, beginning 2 mm from the anterior tip of the frontal lobe from the right and left hemispheres, approximately 5 mm were dissected, which corresponded to the intralesional and contralateral regions [25, 29, 50].

### Neurological evaluation and TTC stain

The neurological scores were evaluated after reperfusion for 24 h. Scoring was performed blindly by a third experimenter using Longa’s neurological scoring system [49, 51]. To confirm neuroprotective effects, the ischemic lesion was measured using TTC stain [52]. Briefly, the animal brains were rapidly removed, frozen immediately at − 80 °C for 5 min, and then sectioned into 2-mm coronal slices. The sections were stained with 2% TTC in PBS at 55 °C for 20 min and fixed in 4% paraformaldehyde (PFA). The infarct volume was measured using Image-Pro Plus image analysis software.

### Cell culture and exosome isolation

Mesenchymal stem cells were derived from mouse bone marrow tibias and femurs and cultured in low-glucose Dulbecco’s modified Eagle’s medium (DMEM) containing 10% fetal bovine serum (FBS) (Gibco) without exosomes (FBS was centrifuged at 200,000*g* for 18 h to deplete exosomes) and then incubated at 37 °C in 5% CO_2_. To label exosomes with tdTomato, cells were stably transduced with packaged lentivirus vectors to express tdTomato fused with the palmitoylation sequence of growth cone-associated protein (PalmtdTomato). The plasmid was kindly provided by Dr Bakhos Tannous (Massachusetts General Hospital, Boston, MA, USA). The harvested supernatants were collected to isolate exosomes according to a previous study [53]. The supernatant was centrifuged at 1000*g* for 30 min followed by 10,000*g* for 30 min at 4 °C to remove cells and debris and then was centrifuged at 140,000*g* for 90 min at 4 °C in a Type Ti70 rotor using an L-80XP ultracentrifuge (Beckman). After resuspension in PBS, the exosome pellet was ultracentrifuged again for 90 min at 140,000*g*. Finally, the exosomes were resuspended in PBS, filtered using a 0.22-μm filter (Millipore), and analyzed with a Micro BCA Protein Assay kit (Pierce).

### Ligand conjugation and microRNA incorporation

Briefly, 0.5 mg/mL exosomes in PBS was reacted with 3 μM dibenzocyclooctyne-sulfo-*N*-hydroxysuccinimidylester (DBCO-NHS) (Sigma) on a rotating mixer at room temperature (RT) for 4 h. Then, the mixture was washed three times using 100-kDa ultrafiltration tubes (Millipore) to remove unconjugated DBCO-NHS. Then, the collected DBCO-conjugated exosomes were ready for linkage to azide-containing molecules. According to the manufacturer, c(RGDyK) peptides with an azide group were synthetized by conjugating 5-azidopentanoic acid to the side chain of lysine (ChinaPeptides). Subsequently, 0.3 μM c(RGDyK) with azide was added to DBCO-conjugated exosomes, and 0.3 μM Cy5.5-azide (Lumiprobe) was added if needed. The reaction was conducted for 12 h at 4 °C on a rotating mixer. Then, the RGD-exo were floated on a 30% sucrose/D2O cushion and centrifuged at 164,000*g* for 90 min using an SW41Ti rotor (Beckman Coulter) to remove unincorporated ligands. After washing with PBS, the modified exosomes were resuspended and stored. As a control, scrambled c(RDGyK) peptides were conjugated to exosomes (Scr-exo).

miR-210 and NC were synthesized with cholesterol conjugated on the 3′ terminus and modified with 2′ Ome (GenePharma). The sequences were as follows: 5′-CUGUGCGUGUGACAHCHHCUGAAGCCGCUGUCACACGCACAGUU-3′ for miR-210, 5′-UUCUCCGAACGUGUCACGUTTACGUGACACGUUCGGAGAATT-3′ for NC. Then, 100 nM cholesterol-conjugated miR-210 was incubated with 100 μg RGD-exo in 200 μL of PBS at 37 °C for 1 h. miR-210 inserted into the exosome membrane through a hydrophobic interaction. After washing with PBS at 140,000*g* for 90 min, the modified exosomes were resuspended and stored at − 80 °C prior to use.

### TEM, NTA and NIRF imaging

Exosomes were observed with a Tecnai G2 transmission electron microscope (FEI). Samples were fixed with 1% glutaraldehyde, applied onto a carbon-coated copper grid, and stained with 1% phosphotungstic acid. NTA was performed using a ZetaView system (Particle Metrix) to track the Brownian motion of exosomes suspended in PBS, and size distribution data was generated by applying the Stokes–Einstein equation. For NIRF imaging, an IVIS spectrum imaging system (PerkinElmer) was used to detect the Cy5.5 fluorescence signals in organs.

### Exosome administration and BrdU labeling

Each mouse was administered 100 µg RGD-exo in 0.2 mL PBS via the tail vein 24 h after reperfusion. PBS or Scr-exo were injected as controls. The mice were sacrificed and dissected 6 h later, and NIRF imaging and immunofluorescence was performed. To deliver miR-210 to the ischemic region, 100 μg RGD-exo:miR-210 were administered 24 h after reperfusion. RGD-exo:NC were injected as a control. The level of miR-210 was examined 12 h later, and the VEGF mRNA level was analyzed 24 h later. To explore the long-term therapeutic effects, the mice were intravenously injected with 100 µg RGD-exo:miR-210 or RGD-exo:NC once every other day. To observe cell proliferation, on the 1st to 7th days after MCAO/R, BrdU (50 mg/kg in saline) was injected intraperitoneally every day. For the sham group, the mice were injected with the same dose of BrdU on the same days after the sham operation.

### Western blotting

The tissues corresponding to the lesion region were dissected 24 h after exosome administration for Western blotting. To detect exosome markers and the VEGF level, Western blotting was carried out as follows. The lesion region in the brains from each group was homogenized with lysis buffer. After centrifugation at 4000 rpm and 4 °C for 15 min, an equal amount (40 μg) of the supernatant of extracted protein samples was loaded and separated on sodium dodecyl sulfate polyacrylamide gels and then transferred onto 0.4-μm PVDF membranes (Millipore). Blocking was performed with 5% skim milk for 1 h at RT, and the membranes were incubated with primary antibodies against Alix (1:1000, Abcam), TSG101 (1:1000, Abcam), Calnexin (1:1000, Abcam), VEGF (1:1000, Proteintech), and tubulin (1:1000, Bioworld Technology) overnight at 4 °C, followed by incubation with secondary antibodies for 1 h at RT. Protein bands were finally visualized using High-sig ECL Western Blotting Substrate (Tianon). The expression level of the proteins was analyzed using ImageJ software.

### Immunofluorescence staining and confocal imaging

Mice were anesthetized and perfused with cold PBS, followed by 4% PFA in PBS (pH 7.4). The brains were dissected, kept in 4% PFA in PBS overnight followed by 30% sucrose in PBS for 48 h, and then cry sectioned at a thickness of 40 μm. The sections were treated with 0.3% Triton-100 for 30 min, blocked with 3% BSA for 2 h, and then immunostained with anti-Integrin β_3_ (1:500, Santa Cruz) and anti-CD34 (1:400, Abcam) antibodies overnight at 4 °C. After 5 washes with PBST (PBS containing 0.1% Triton-100), the samples were incubated with FITC- or Alexa 647-conjugated secondary antibodies (Invitrogen) for 1 h at RT. For BrdU staining, brain sections from the BrdU labeled mice and sham-operated mice were collected. After another 5 washes with PBST and staining with DAPI, with post-fix in 4% PFA for 10 min, slices were incubated in HCl (2 mol/L) at 37 °C for 15 min. Then washed them in PBS 3 times for 5 min each, and block in 0.1% Triton-X and 5% BSA for 1 h at RT. Then immunostained with anti-BrdU (1:250, Abcam) overnight at 4 °C. After 5 washes with PBST, the samples were incubated with FITC-conjugated secondary antibodies (1:500, Proteintech) for 1 h at RT. After another 5 washes with PBST and staining with DAPI, the slices were imaged with an FV-1200 confocal microscope (Olympus). Images were processed and analyzed using ImageJ software (NIH).

### Quantitative real-time PCR

Twelve hours after administration, total RNA from the lesion tissues was extracted with Trizol reagent (Invitrogen). cDNA synthesis was performed using a PrimeScript RT reagent Kit (Takara). RT-PCR reactions were carried out on a Lightcycler 96 system (Roche) in 10 μL reactions with 1 μL of cDNA samples using SYBR mix (Vazyme Biotech). The primer sequences were as follows: 5′-TTACTGCTGTACCTCCACC-3′ (forward) and 5′-ACAGGACGGCTTGAAGATG-3′ (reverse) for VEGF. The miR-210 and U6 primers were applied using Bulge-Loop miRNA qRT-PCR primer (RiboBio). Relative expression was calculated by the comparative 2^−ΔΔCt^ method. All experiments were performed at least three times independently.

### Statistical analysis

The data are presented as the mean ± SEM. Statistical analysis was accomplished using GraphPad Prim software (GraphPad Software). Comparisons between two groups were performed with Student’s *t*-test. Significant differences among multiple groups were determined by one-way analysis of variance (ANOVA) or two-way ANOVA followed by Tukey’s post hoc test. *P* values < 0.05 were considered statistically significant.

## Additional files

**Additional file 1: Figure S1.** Estimation of the number of c(RGDyK) peptide, Cy5.5, or miR-210 incorporated onto the exosomes. **a** Black squares show fluorescent intensities of c(RK(FITC)DyK) standard curve at 100-800 nM. **b** The average concentration of RGD is 116 pmol / 10^11^ particles on exosomes according to the standard curve. **c** Black squares show fluorescent intensities of FITC-miR-210 at concentrations of 50-500 nM. **d** The average concentrations of miR-210 loaded with Exo:miR-210, Scr-exo:miR-210, or RGD-exo:miR-210 are 118, 110, or 108 pmol / 10^11^ particles calculated according to the standard curve. **e** Black squares show fluorescent intensities of Cy5.5 at concentrations of 100-800 nM. **f** The average concentrations of labeled Cy5.5 on the Exo, Scr-exo, or RGD-exo are 71, 67, or 65 pmol / 10^11^ particles according to the standard curve.

**Additional file 2: Figure S2.** RGD**-**exo colocalized with CD34 in brain after injection. **a, b** Co-labelled fluorescence images of RGD-exo (red) and CD34 (green) in the ischemic cortex 6 h after intravenous administration of tdTomato-labeled RGD-exo on the mice receiving MCAO/R or Sham. Blue indicates nuclei, and CD34 was marked by green. A magnification indicated the co-localization of RGD-exo and CD34.

**Additional file 3: Figure S3.** RGD**-**exo:miR-210 increased endothelia cells proliferation after 7 days of reperfusion. Double staining of BrdU (green) and CD34 (red) after RGD-exo:NC or RGD-exo:miR-210 injection in the ischemic brain.

## Abbreviations

miRNA or miR: micro ribonucleic acid
miR-210: microRNA-210
MCAO: middle cerebral artery occlusion
VEGF: vascular endothelial growth factor
BBB: blood–brain barrier
EVs: extracellular vesicles
mRNA: messenger ribonucleic acid
RGD-exo: cyclo (Arg-Gly-Asp-D-Tyr-Lys) peptide [c(RGDyK)] conjugated exosomes
siRNA: small interfering ribonucleic acid
MSC: mesenchymal stromal cell
MCAO/R: MCAO and reperfusion
TTC: 2,3,5-triphenyltetrazolium chloride
kDa: kilodalton
NIRF: near-infrared fluorescence
TEM: transmission electron microscopy
NTA: nanoparticle tracking analysis
PBS: phosphate-buffered saline
PFA: paraformaldehyde
DMEM: Dulbecco’s modified Eagle’s medium
FBS: fetal bovine serum
DBCO-NHS: dibenzocyclooctyne-sulfo-*N*-hydroxysuccinimidyl ester
RT: room temperature
BrdU: 5-bromo-2-deoxyuridine
NC: negative control
FEI: transmission electron microscope
FITC: fluorescein isothiocyanate
Cy5.5: cyanine 5.5 far-red fluorescent dye
ASC: adipose-derived stem cell
ISCU2: iron–sulfur cluster scaffold homolog 2
PTPN2: protein tyrosine phosphatase nonreceptor type 2
SEM: standard error of the mean
ANOVA: analysis of variance

## References

[1] Neuhaus AA, Couch Y, Hadley G, Buchan AM (2017). Neuroprotection in stroke: the importance of collaboration and reproducibility. Brain.

[2] Bai YY, Gao X, Wang YC, Peng XG, Chang D, Zheng S, Li C, Ju S (2014). Image-guided pro-angiogenic therapy in diabetic stroke mouse models using a multi-modal nanoprobe. Theranostics..

[3] Abumiya T, Fitridge R, Mazur C, Copeland BR, Koziol JA, Tschopp JF, Pierschbacher MD, del Zoppo GJ (2000). Integrin alpha(IIb)beta(3) inhibitor preserves microvascular patency in experimental acute focal cerebral ischemia. Stroke.

[4] Di Y, Lei Y, Yu F, Changfeng F, Song W, Xuming M (2014). MicroRNAs expression and function in cerebral ischemia reperfusion injury. J Mol Neurosci.

[5] Ambros V (2004). The functions of animal microRNAs. Nature.

[6] Bartel DP (2004). MicroRNAs: genomics, biogenesis, mechanism, and function. Cell.

[7] He L, Hannon GJ (2004). MicroRNAs: small RNAs with a big role in gene regulation. Nat Rev Genet.

[8] Fasanaro P, Greco S, Lorenzi M, Pescatori M, Brioschi M, Kulshreshtha R, Banfi C, Stubbs A, Calin GA, Ivan M (2009). An integrated approach for experimental target identification of hypoxia-induced miR-210. J Biol Chem.

[9] Meng ZY, Kang HL, Duan W, Zheng J, Li QN, Zhou ZJ (2018). MicroRNA-210 promotes accumulation of neural precursor cells around ischemic foci after cerebral ischemia by regulating the SOCS1-STAT3-VEGF-C pathway. J Am Heart Assoc.

[10] Lou YL, Guo F, Liu F, Gao FL, Zhang PQ, Niu X, Guo SC, Yin JH, Wang Y, Deng ZF (2012). miR-210 activates notch signaling pathway in angiogenesis induced by cerebral ischemia. Mol Cell Biochem.

[11] Zeng L, Liu J, Wang Y, Wang L, Weng S, Tang Y, Zheng C, Cheng Q, Chen S, Yang GY (2011). MicroRNA-210 as a novel blood biomarker in acute cerebral ischemia. Front Biosci (Elite Ed)..

[12] Hu S, Huang M, Li Z, Jia F, Ghosh Z, Lijkwan MA, Fasanaro P, Sun N, Wang X, Martelli F (2010). MicroRNA-210 as a novel therapy for treatment of ischemic heart disease. Circulation.

[13] Liu F, Lou YL, Wu J, Ruan QF, Xie A, Guo F, Cui SP, Deng ZF, Wang Y (2012). Upregulation of MicroRNA-210 regulates renal angiogenesis mediated by activation of VEGF signaling pathway under ischemia/perfusion injury in vivo and in vitro. Kidney Blood Pressure Res.

[14] Zeng LL, He XS, Liu JR, Zheng CB, Wang YT, Yang GY (2016). Lentivirus-mediated overexpression of MicroRNA-210 improves long-term outcomes after focal cerebral ischemia in mice. CNS Neurosci Ther.

[15] Zeng L, He X, Wang Y, Tang Y, Zheng C, Cai H, Liu J, Wang Y, Fu Y, Yang GY (2014). MicroRNA-210 overexpression induces angiogenesis and neurogenesis in the normal adult mouse brain. Gene Ther.

[16] Cook DJ, Teves L, Tymianski M (2012). Treatment of stroke with a PSD-95 inhibitor in the gyrencephalic primate brain. Nature.

[17] Thompson BJ, Ronaldson PT (2014). Drug delivery to the ischemic brain. Adv Pharmacol.

[18] Rufino-Ramos D, Albuquerque PR, Carmona V, Perfeito R, Nobre RJ, Pereira de Almeida L (2017). Extracellular vesicles: novel promising delivery systems for therapy of brain diseases. J Control Release.

[19] Wang W, Luo J, Wang S (2018). Recent progress in isolation and detection of extracellular vesicles for cancer diagnostics. Adv Healthcare Mater.

[20] Ahmed M, Carrascosa LG, Wuethrich A, Mainwaring P, Trau M (2018). An exosomal- and interfacial-biosensing based strategy for remote monitoring of aberrantly phosphorylated proteins in lung cancer cells. Biomater Sci..

[21] Bunggulawa EJ, Wang W, Yin T, Wang N, Durkan C, Wang Y, Wang G (2018). Recent advancements in the use of exosomes as drug delivery systems. J Nanobiotechnol.

[22] Alyautdin R, Khalin I, Nafeeza MI, Haron MH, Kuznetsov D (2014). Nanoscale drug delivery systems and the blood-brain barrier. Int J Nanomed.

[23] Quek C, Hill AF (2017). The role of extracellular vesicles in neurodegenerative diseases. Biochem Biophys Res Commun.

[24] Yong X, Yang X, Emory SR, Wang J, Dai J, Yu X, Mei L, Xie J, Ruan G (2018). A potent, minimally invasive and simple strategy of enhancing intracellular targeted delivery of Tat peptide-conjugated quantum dots: organic solvent-based permeation enhancer. Biomater Sci.

[25] Tian T, Zhang HX, He CP, Fan S, Zhu YL, Qi C, Huang NP, Xiao ZD, Lu ZH, Tannous BA, Gao J (2018). Surface functionalized exosomes as targeted drug delivery vehicles for cerebral ischemia therapy. Biomaterials.

[26] Alvarez-Erviti L, Seow YQ, Yin HF, Betts C, Lakhal S, Wood MJA (2011). Delivery of siRNA to the mouse brain by systemic injection of targeted exosomes. Nat Biotechnol.

[27] Haney MJ, Klyachko NL, Zhaoa YL, Gupta R, Plotnikova EG, He ZJ, Patel T, Piroyan A, Sokolsky M, Kabanov AV, Batrakova EV (2015). Exosomes as drug delivery vehicles for Parkinson’s disease therapy. J Control Release.

[28] O’Loughlin AJ, Mager I, de Jong OG, Varela MA, Schiffelers RM, El Andaloussi S, Wood MJA, Vader P (2017). Functional delivery of lipid-conjugated siRNA by extracellular vesicles. Mol Ther.

[29] Zhu DY, Deng Q, Yao HH, Wang DC, Deng Y, Liu GQ (2002). Inducible nitric oxide synthase expression in the ischemic core and penumbra after transient focal cerebral ischemia in mice. Life Sci.

[30] Zaccagnini G, Maimone B, Fuschi P, Maselli D, Spinetti G, Gaetano C, Martelli F (2017). Overexpression of miR-210 and its significance in ischemic tissue damage. Sci Rep.

[31] Zaccagnini G, Maimone B, Di Stefano V, Fasanaro P, Greco S, Perfetti A, Capogrossi MC, Gaetano C, Martelli F (2014). Hypoxia-induced miR-210 modulates tissue response to acute peripheral ischemia. Antioxid Redox Signal.

[32] Didiot MC, Hall LM, Coles AH, Haraszti RA, Godinho BM, Chase K, Sapp E, Ly S, Alterman JF, Hassler MR (2016). Exosome-mediated delivery of hydrophobically modified siRNA for huntingtin mRNA silencing. Mol Ther.

[33] Lai CP, Kim EY, Badr CE, Weissleder R, Mempel TR, Tannous BA, Breakefield XO (2015). Visualization and tracking of tumour extracellular vesicle delivery and RNA translation using multiplexed reporters. Nat Commun.

[34] Tian YH, Li SP, Song J, Ji TJ, Zhu MT, Anderson GJ, Wei JY, Nie GJ (2014). A doxorubicin delivery platform using engineered natural membrane vesicle exosomes for targeted tumor therapy. Biomaterials.

[35] Ley K, Rivera-Nieves J, Sandborn WJ, Shattil S (2016). Integrin-based therapeutics: biological basis, clinical use and new drugs. Nat Rev Drug Discov..

[36] Li L, Welser JV, Milner R (2010). Absence of the alpha v beta 3 integrin dictates the time-course of angiogenesis in the hypoxic central nervous system: accelerated endothelial proliferation correlates with compensatory increases in alpha 5 beta 1 integrin expression. J Cereb Blood Flow Metab.

[37] Rosenstein JM, Mani N, Silverman WF, Krum JM (1998). Patterns of brain angiogenesis after vascular endothelial growth factor administration in vitro and in vivo. Proc Natl Acad Sci USA.

[38] Agrawal R, Pandey P, Jha P, Dwivedi V, Sarkar C, Kulshreshtha R (2014). Hypoxic signature of microRNAs in glioblastoma: insights from small RNA deep sequencing. BMC Genomics..

[39] Qiu J, Zhou XY, Zhou XG, Cheng R, Liu HY, Li Y (2013). Neuroprotective effects of microRNA-210 on hypoxic-ischemic encephalopathy. Biomed Res Int.

[40] Liu LL, Li D, He YL, Zhou YZ, Gong SH, Wu LY, Zhao YQ, Huang X, Zhao T, Xu L (2017). miR-210 protects renal cell against hypoxia-induced apoptosis by targeting HIF-1 alpha. Mol Med.

[41] Kim JH, Park SG, Song SY, Kim JK, Sung JH (2013). Reactive oxygen species-responsive miR-210 regulates proliferation and migration of adipose-derived stem cells via PTPN2. Cell Death Dis..

[42] Ruan L, Wang B, ZhuGe Q, Jin K (2015). Coupling of neurogenesis and angiogenesis after ischemic stroke. Brain Res.

[43] Kwon EJ, Skalak M, Lo BuR, Bhatia SN (2016). Neuron-targeted nanoparticle for siRNA delivery to traumatic brain injuries. ACS Nano.

[44] Sun L, Xu R, Sun X, Duan Y, Han Y, Zhao Y, Qian H, Zhu W, Xu W (2016). Safety evaluation of exosomes derived from human umbilical cord mesenchymal stromal cell. Cytotherapy..

[45] Zhang ZG, Chopp M (2016). Exosomes in stroke pathogenesis and therapy. J Clin Invest..

[46] Kooijmans SAA, Stremersch S, Braeckmans K, de Smedt SC, Hendrix A, Wood MJA, Schiffelers RM, Raemdonck K, Vader P (2013). Electroporation-induced siRNA precipitation obscures the efficiency of siRNA loading into extracellular vesicles. J Control Release.

[47] Cocucci E, Racchetti G, Meldolesi J (2009). Shedding microvesicles: artefacts no more. Trends Cell Biol.

[48] Todorova D, Simoncini S, Lacroix R, Sabatier F, Dignat-George F (2017). Extracellular vesicles in angiogenesis. Circ Res.

[49] Longa EZ, Weinstein PR, Carlson S, Cummins R (1989). Reversible middle cerebral artery occlusion without craniectomy in rats. Stroke.

[50] Ashwal S, Tone B, Tian HR, Cole DJ, Pearce WJ (1998). Core and penumbral nitric oxide synthase activity during cerebral ischemia and reperfusion. Stroke..

[51] Shimamura N, Matchett G, Tsubokawa T, Ohkuma H, Zhang J (2006). Comparison of silicon-coated nylon suture to plain nylon suture in the rat middle cerebral artery occlusion model. J Neurosci Methods.

[52] Bederson JB, Pitts LH, Germano SM, Nishimura MC, Davis RL, Bartkowski HM (1986). Evaluation of 2,3,5-triphenyltetrazolium chloride as a stain for detection and quantification of experimental cerebral infarction in rats. Stroke.

[53] Thery C, Amigorena S, Raposo G, Clayton A (2006). Isolation and characterization of exosomes from cell culture supernatants and biological fluids. Curr Protoc Cell Biol..

