# Supplementary figures and images for: Exosome-mediated targeted delivery of miR-210 for angiogenic therapy after cerebral ischemia in mice
Source: J Nanobiotechnology. 2019 Feb 19;17:29. doi: 10.1186/s12951-019-0461-7 (PMC6379944; doi:10.1186/s12951-019-0461-7)

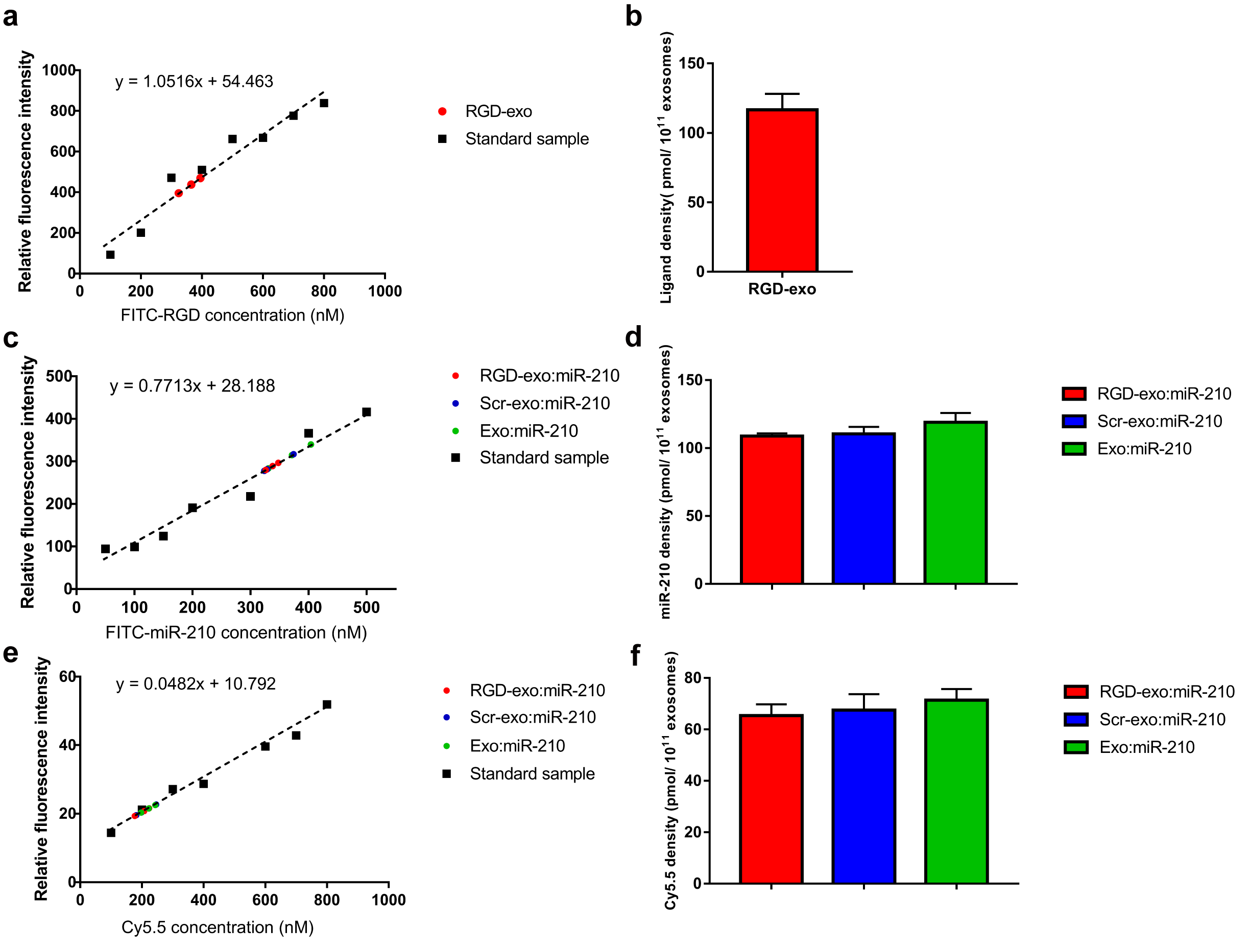

Supplement: Supplementary file 1 — Additional file 1: Figure S1. Estimation of the number of c(RGDyK) peptide, Cy5.5, or miR-210 incorporated onto the exosomes. a Black squares show fluorescent intensities of c(RK(FITC)DyK) standard curve at 100-800 nM. b The average concentration of RGD is 116 pmol / 1011 particles on exosomes according to the standard curve. c Black squares show fluorescent intensities of FITC-miR-210 at concentrations of 50-500 nM. d The average concentrations of miR-210 loaded with Exo:miR-210, Scr-exo:miR-210, or RGD-exo:miR-210 are 118, 110, or 108 pmol / 1011 particles calculated according to the standard curve. e Black squares show fluorescent intensities of Cy5.5 at concentrations of 100-800 nM. f The average concentrations of labeled Cy5.5 on the Exo, Scr-exo, or RGD-exo are 71, 67, or 65 pmol / 1011 particles according to the standard curve. [file 12951_2019_461_MOESM1_ESM.tif]

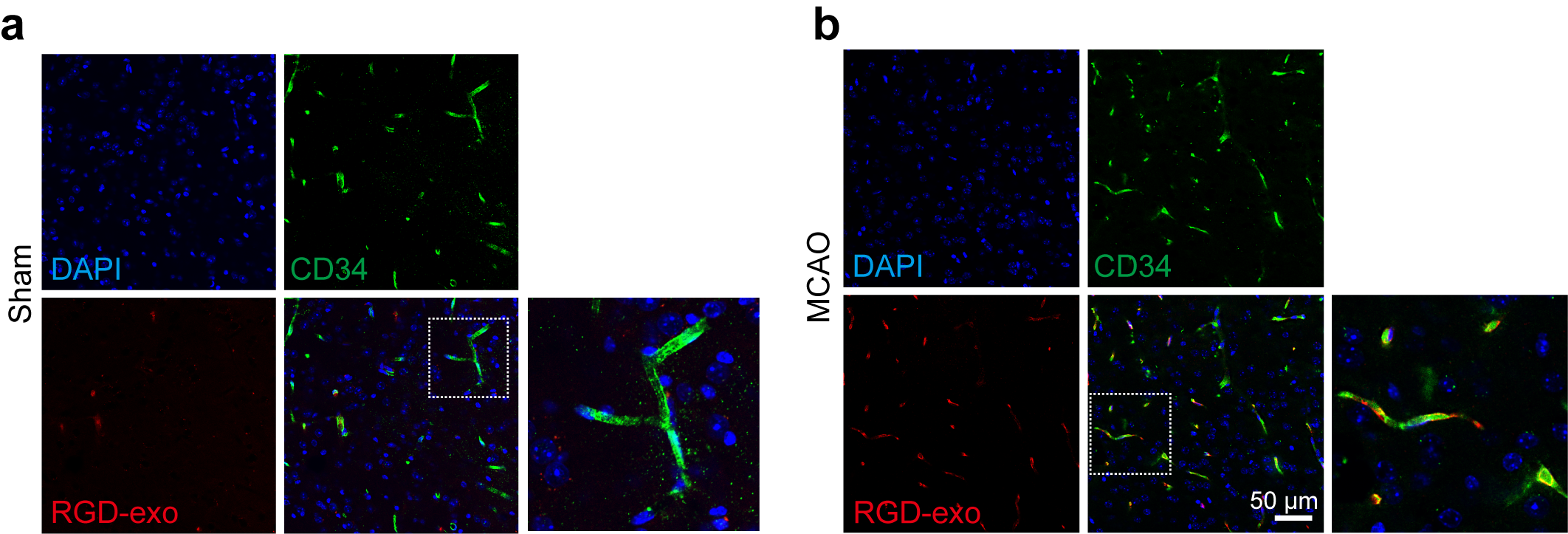

Supplement: Supplementary file 2 — Additional file 2: Figure S2. RGD-exo colocalized with CD34 in brain after injection. a, b Co-labelled fluorescence images of RGD-exo (red) and CD34 (green) in the ischemic cortex 6 h after intravenous administration of tdTomato-labeled RGD-exo on the mice receiving MCAO/R or Sham. Blue indicates nuclei, and CD34 was marked by green. A magnification indicated the co-localization of RGD-exo and CD34. [file 12951_2019_461_MOESM2_ESM.tif]

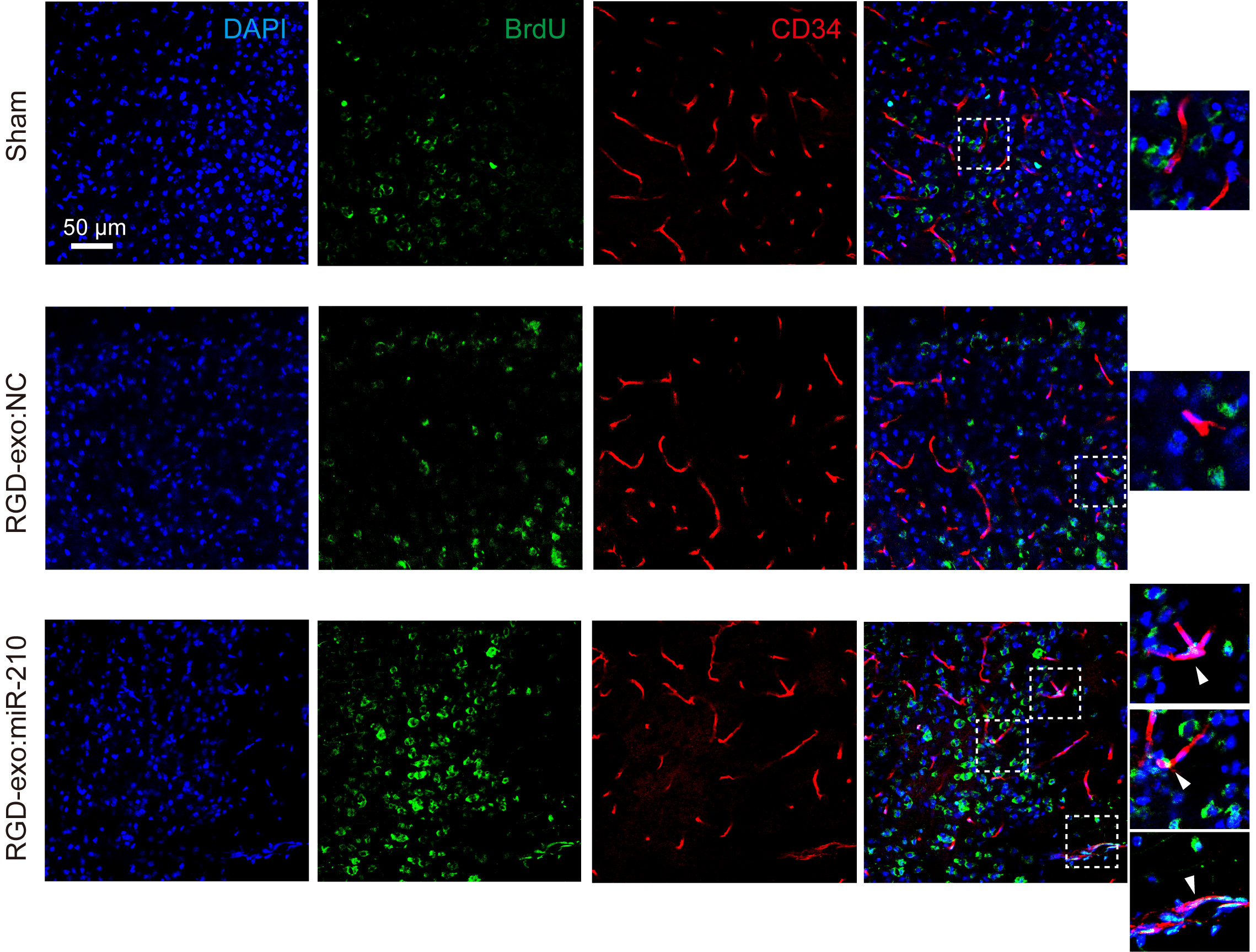

Supplement: Supplementary file 3 — Additional file 3: Figure S3. RGD-exo:miR-210 increased endothelia cells proliferation after 7 days of reperfusion. Double staining of BrdU (green) and CD34 (red) after RGD-exo:NC or RGD-exo:miR-210 injection in the ischemic brain. [file 12951_2019_461_MOESM3_ESM.tif]
